# Administration of the GLP-1 receptor agonist exenatide in rats improves functional recovery after spinal cord injury by reducing endoplasmic reticulum stress

**DOI:** 10.1016/j.ibneur.2023.09.003

**Published:** 2023-09-11

**Authors:** Satoshi Nomura, Hiroyuki Katoh, Sho Yanagisawa, Toshihiro Noguchi, Keiko Okada, Masahiko Watanabe

**Affiliations:** Department of Orthopaedic Surgery, Surgical Science, Tokai University School of Medicine, 143 Shimokasuya, Isehara, Kanagawa 259-1193, Japan

**Keywords:** Spinal cord injury, Endoplasmic reticulum stress, Glucagon-like peptide-1 (GLP-1), Glucose-regulated protein78 (GRP78), C/EBP homologous transcription factor protein (CHOP)

## Abstract

After spinal cord injury (SCI), endoplasmic reticulum (ER) stress has been reported to be an integral part of the secondary injury process that causes apoptosis of glial cells, leading to remyelination failure. This report focuses on exenatide, a glucagon-like peptide-1 (GLP-1) receptor agonist widely used to treat diabetes, as a potential agent to improve functional outcome after SCI by improving the ER stress response. Exenatide administered subcutaneously immediately after injury and 7 days later in a rat model of moderate contusive SCI revealed significant improvement in hindlimb function without any hypoglycemia. Changes in the expression of glucose regulatory protein 78 (GRP78), an endoplasmic reticulum chaperone that protects against ER stress, and C/EBP homologous transcription factor protein (CHOP), a pro-apoptotic transcription factor in the apoptosis pathway were examined as indices of ER stress. We found that administration of exenatide after SCI suppressed CHOP while increasing GRP78 in the injured spinal cord, leading to a significant decrease in tissue damage and a significant increase in oligodendrocyte progenitor cell survival. This study suggests that administration of exenatide after SCI decreases ER stress and improves functional recovery without any apparent side-effects.

## Introduction

Spinal cord injury (SCI) is a devastating condition that leads to life-long physical and mental distress, and there is currently no established treatment. The pathophysiological events of traumatic SCI are twofold: the primary injury resulting from the direct physical impact, and the subsequent secondary injury that occurs as a consequence of the primary injury and expands the lesion area due to biochemical and vascular sequelae (Lu et al., 2000, Tator and Fehlings, 1991, Watanabe et al., 1998). Recent studies have reported on endoplasmic reticulum (ER) stress as an integral part of the secondary injury process, leading to apoptosis of oligodendrocyte precursor cells (OPCs) after SCI. Although OPC proliferate around the injury site after SCI, most of these cells disappear without differentiating into mature oligodendrocytes through apoptosis (Ishii et al., 2001, McTigue et al., 2001). The apoptosis of OPC causes further functional deterioration after SCI due to demyelination of the remaining nerve fibers, and also inhibits subsequent spinal cord regeneration due to remyelination failure. In contrast, astrocytes proliferate around the injury site and forms a glial scar without undergoing apoptosis (Baldwin et al., 1998, Eng et al., 2000, Fawcett and Asher, 1999, McKeon et al., 1991). One of the reasons for the difference in survival between OPCs and astrocytes stemmed from the difference in the ER stress response between these two cell types (Matsuyama et al., 2014).

When cells encounter stress such as electrolyte imbalance or increased excitatory amino acids, the normal folding of translated proteins within the ER is disrupted. This leads to the accumulation of unfolded or misfolded proteins in the ER in a condition that has been aptly termed ER stress (Boyce and Yuan, 2006, Larner et al., 2006, Schröder and Kaufman, 2005). This triggers the activation of the ER stress response, which is a process that is instigated to decrease ER stress in the cell. The cells’ response to increased ER stress is the unfolded protein response (UPR), in which the ER chaperone glucose-regulated protein 78 (GRP78) plays a major part to decrease ER stress and prevent apoptosis (Bertolotti et al., 2000, Haze et al., 1999, Ron and Walter, 2007, Shen et al., 2002). The UPR inhibits translation to attenuate protein synthesis and increases the production of molecular chaperones involved in protein folding. However, when the UPR is overwhelmed by increasing ER stress, apoptosis mediated by C/EBP homologous transcription factor protein (CHOP) ensues (Oyadomari and Mori, 2004, Woehlbier and Hetz, 2011, Zinszner et al., 1998).

The cellular response and unfolded protein response to this ER stress have been reported in SCI (Ohri et al., 2011, Penas et al., 2007), but there are many unknown aspects of the detailed mechanism within the injured spinal cord. The effect of ER stress in cellular homeostasis was analyzed through the expression of GRP78 and CHOP as indices of the ER stress response. When C6 glioma cells were virally transfected to express GRP78 and cultured with the ER stress-inducer tunicamycin, cells expressing GRP78 had significantly lower levels of apoptosis compared to control C6 cells, demonstrating the cytoprotective effect of GRP78 (Suyama et al., 2011). The effect of modulating the ER stress response was explored by administrating amiloride, a potassium-conserving diuretic that has been shown to be neuroprotective against multiple sclerosis (Friese et al., 2007) and Parkinson’s disease (Arias et al., 2008). Thoracic spinal cord contusive SCI rats that received intraperitoneally administration of amiloride had increased expression of GRP78 and decreased expression of CHOP, leading to significantly lower apoptotic cells, greater levels of myelination, and improved hindlimb motor function compared to control animals (Kuroiwa et al., 2014). However, amiloride is a diuretic used to lower blood pressure and is therefore contraindicated in SCI patients immediately after injury. Therefore, an alternate drug to improve the ER stress response without amiloride’s associated fluctuations in blood pressure was sought.

Exenatide, a glucagon-like peptide-1 (GLP-1) receptor agonist widely used as an antidiabetic drug, has also been reported to reduce ER stress in multiple systems such as the pancreas beta cells, joint chondrocytes, and hyperglycemic cardiomyocytes and has been effective in treating amyotrophic lateral sclerosis (ALS), multiple sclerosis, and traumatic encephalopathy (Erbil et al., 2019, Hakon et al., 2015, Hölscher, 2012, Li et al., 2012). Although the therapeutic effects of injecting exenatide intraperitonally after SCI has been reported, the effects of subcutaneously administered exenatide on the ER stress in the injured spinal cord has not been studied. Therefore, the efficacy of exenatide administration as a treatment for SCI was investigated in this study, especially focusing on its effect on ER stress.

## Materials and methods

### Animal model

All animal procedures were performed in accordance with the National Institute of Health Guidelines for the Care and Use of Laboratory Animals and were approved by the Animal Experimentation Committee at Tokai University School of Medicine. Female Sprague-Dawley (SD) rats (9–10 weeks old, weighing 280–320 g) were purchased from Nippon Crea (Kanagawa, Japan) and used for the experiment. Surgery was performed with 4% isoflurane inhalation anesthesia under aseptic conditions. After laminectomy of the 10th thoracic vertebra, the dura was exposed, and a contusive SCI was induced with a force of 200 Kdyne and dwell time of 0 s using an Infinite Horizon spinal cord impactor device (IH impactor: Precision Systems & Instrumentation [PSI], Lexington, KY). The actual force applied to the dura mater was recorded and compared among the exenatide and control groups, confirming that there was no statistical difference between the two groups. Bladder massages were performed twice a day until voluntary urination was restored.

The injured rats were divided into three groups: exenatide, control, and sham groups (n = 10 per group). The exenatide group received 10 µg subcutaneous injection (Exenatide, AstraZeneca, D04121) immediately after injury and at day 7 after injury, while the control group received phosphate buffered saline (PBS) injection and the sham surgery group received a laminectomy without a spinal cord contusion procedure.

Hindlimb motor function was evaluated using the Basso, Beattie, and Breanahan locomotor rating scale (BBB scale) before injury, the day following surgery, and every other day until day 14 after surgery. The evaluation was performed between 9 am and 12 noon by 3 researchers blinded to the procedure, and the mean values of 5-minute observations were used (n = 10 per group).

### Blood glucose level

Blood glucose levels were measured immediately before the injury, immediately after the injury, 3, 6 and 12 h after the injury and every other day thereafter (n = 10 per group). Approximately 1 ml of blood was collected from the caudal vein using a 20 G needle, and blood glucose was measured using a Medisafe FIT blood glucose meter (Terumo, Tokyo, Japan).

### Western blot

The injured spinal cord was exposed under isoflurane 4% inhalational anesthesia on days 1, 3, 7, and 14 after injury, and a 5 mm section of spinal cord tissue centered on the damage epicenter was resected under a microscope. Immediately after removal, the spinal cord was washed in ice-cold PBS, and processed with Cell Lytic NuCLEAR extraction kit (Sigma-Aldrich, St. Louis, MO, USA). 20 µg of spinal cord lysate was loaded into each lane of a 10% SDS polyacrylamide gel, subjected to electrophoreses, and electrotransferred onto nitrocellulose membranes (Bio-Rad, Hercules, CA). The membranes were blocked with 5% BSA in TBST (50 mM Tris, pH 7.6, 150 mM NaCl, 0.1% Tween 20), then incubated overnight at 4 °C with rabbit anti-GRP78 (1:2000; AC-RIS, Rockville, MD, USA) and rabbit anti-CHOP (1:200; Bioworld, Louis Park, MN, USA) antibodies. The membranes were washed in 0.05% Twin-20 in PBS for 7 h, then incubated for 60 min at 25 °C with horse radish peroxidase (HRP)-linked anti-Rabbit IgG (GRP78, 1:5000; CHOP, 1:1000; DAKO, Santa Clara, CAL, USA). The proteins were labeled with Immobilon Western Chemiluminescent HRP (Millipore, Burlington, MA, USA) and analyzed using densitometric scans of the films using the Macintosh software CS analyzer (ATTO, Tokyo, Japan) and normalized against the internal control β-actin labelled with mouse monoclonal antibody against β-actin (1:1000, Sigma-Aldrich, A5441, St. Louis, MO, USA). Expression of GRP78 and CHOP over time was compared between the exenatide group and the control group (n = 5 per group) and presented as a ratio of the mean expression observed in sham animals.

### Histology

Perfusion fixation was performed using 2% paraformaldehyde (PFA) in 0.1 M phosphate buffer (PB) under general anesthesia with isoflurane 4% on days 3, 7, and 14 after injury. After perfusion fixation, the spinal cords were removed and fixed in 2% PFA in 0.1 M PB at 4 ℃ for 2 days, followed by a dehydration process in stages with 7%, 15% and 20% sucrose water. The spinal cords were embedded and frozen in Optimal Cutting Temperature (OCT) compound (Sakura Finetek, Tokyo, Japan), and were sectioned at a thickness of 10 µm on a cryostat microtome (CM3050S, Leica Biosystems, USA). A 2 mm area equating to the tip of the IH impactor was set as the epicenter, and sections were prepared at 3 mm intervals, at the epicenter, 4 mm, 7 mm, 10 mm and 13 mm.

Day 14 sections were stained with hematoxylin-eosin (HE) and Luxol fast blue (LFB) (n = 5 per group). For HE staining, sections were washed in deionized water for 5 min and then continuously stained by hematoxlin for 2.5 min and by eosin for 16 s. For Luxol Fast Blue (LFB) staining, sections were stained with a 0.1% LFB (Sigma-Aldrich) solution, sealed, washed with distilled water, and placed in 95% alcohol for 10 min. Each mixture was separated in a 0.05% lithium carbonate aqueous solution for 10 s and in a 70% alcohol solution for 20 s. The above two steps were repeated until the grey and white matter were clearly observable under the microscope. The above sections were dehydrated by a conventional alcohol gradient (80%, 95%, 95%, 100%, 100% alcohol for 2 min), placed in xylene I and II for 10 min, and then sealed with neutral gum. Images were captured with an Olympus Microscopy Europa BX63 (OLYMPUS, Japan), and the lesion cavity and myelinated areas were quantified with cellSens Dimension (OLYMPUS, Japan). The cavity volume was estimated utilizing Cavalieri’s principle, in which the cavity area on each section was multiplied by intersection distance and totaled into a series summation. For the evaluation of LFB-positive myelinated area, the average of three sections per animal were evaluated and presented as a percentage of the total area.

Sections taken 7 mm caudal to the lesion epicenter from spinal cords collected on day 3, 7 and 14 after injury were used for cytological immunohistochemical staining (n = 5 per group). The lesion epicenter was specifically not selected because the damage at the epicenter led to significant loss of cells that made quantification difficult. The sections were washed 3 times for 10 min with PBS, blocked at 24 ℃ for 60 min with 5% normal goat serum in PBS, washed again for 10 min, and then incubated overnight at 4 ℃ with rabbit anti-GRP78 (1:2000; ACRIS, Rockville, MD, USA), rabbit anti-CHOP (1:200; Bioworld, Louis Park, MN, USA), mouse anti-NG2 (1:50, Millipore, Burlington, MA, USA), and mouse anti-GFAP(1:400, Millipore, Burlington, MA, USA). The sections were washed with PBS, then incubated in a darkened room for 60 min at 24 ℃ with the following secondary antibodies: goat anti-rabbit Alexa Fluor 594 (GRP78: 1:200, CHOP: 1:100, Abcam, ab150116, London, UK), goat anti-mouse Alexa Fluor 488 (1:1000, Abcam, ab150077, London, UK). VECTASHIELD with DAPI (Vector Laboratories) was used for nuclear staining and encapsulation and the stained sections were observed with a fluorescence microscope. The total number of NG2-positive OPCs and GFAP-positive astrocytes in the dorsal column of five consecutive sections were counted. The ratios of these cells that were also positive for GRP78 or CHOP were calculated, and the differences between the two groups were compared (n = 5 per group).

### Statistical processing

All data were analyzed using two-way repeated-measures analysis of variance (ANOVA) with Tukey’s post hoc multiple comparison tests. The level of significance was set at p < 0.05.

## Results

### Blood glucose levels

**Fig. 1:**
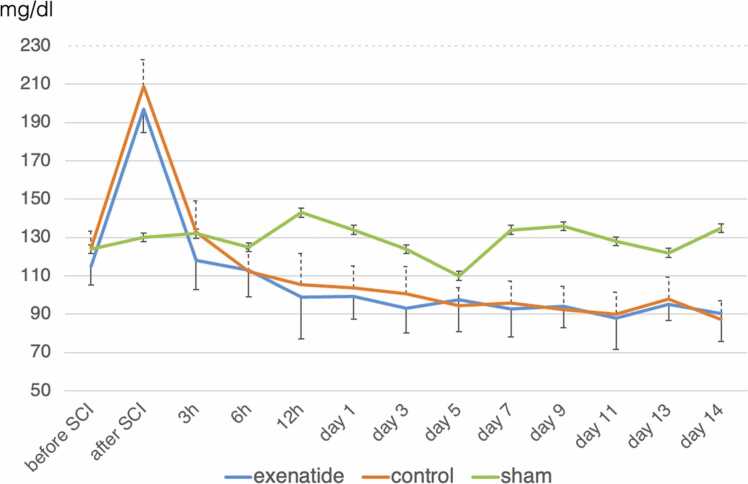
Exenatide administration does not cause hypoglycemia. Blood glucose levels rose sharply immediately after SCI and then gradually descended to approximately 100 mg/dl by 12 h after SCI. No significant difference was observed between the exenatide and control groups, and hypoglycemia was not observed in the exenatide group.

The blood glucose level, which is normally approximately 120 mg/dL in rats, increased to approximately 200 mg/dL in both the exenatide and control groups immediately after injury, but quickly returned to normal levels thereafter. There was no significant difference observed between the two groups and no hypoglycemia was observed in the exenatide group (Fig. 1).

### Western blot of ER stress-related protein expression reveals that exenatide lowers ER stress

**Fig. 2:**
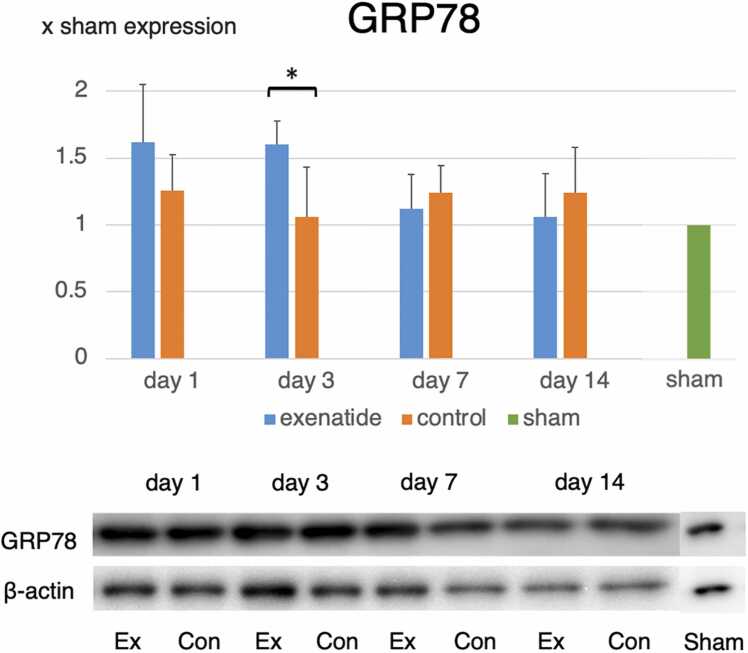
Western blot reveals higher GRP78 expression in exenatide group. Western blot for the ER stress response chaperon GRP78 revealed significantly higher GRP78 expression on day 3, indicating that exenatide administration increases the internal mechanisms to decrease ER stress (p < 0.01, n = 5 per group).

**Fig. 3:**
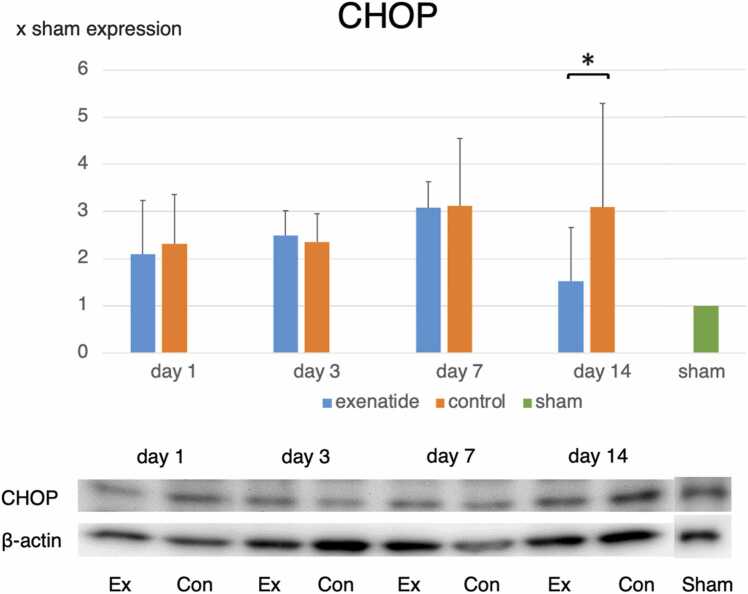
Western blot reveals lower CHOP expression in exenatide group. Western blot for the apoptosis-inducing CHOP GRP78 revealed significantly lower CHOP expression on day 14, indicating that exenatide administration decreased apoptosis-inducing signaling due to ER stress (p < 0.05, n = 5 per group).

The effect of spinal cord injury on ER stress and the effect of exenatide administration were examined by studying the expression of GRP78 and CHOP by western blot. The levels of GRP78, the ER chaperone of the unfolded protein response that enhances the cell’s mechanisms to lower ER stress, were higher in the exenatide group on higher on days 1 and 3 and lower on days 7 and 14, with a significantly higher expression on day 3 after injury (p < 0.05) (Fig. 2). The levels of CHOP, which acts as the trigger for ER stress-related apoptosis when the ER stress is not sufficiently controlled, increased in the exenatide group from day 1 to a peak at day 7, and then decreased thereafter. In the control group, CHOP expression was sustained at high levels at days 7 and 14, with significantly higher CHOP expression compared to the exenatide group at day 14 (p < 0.05) (Fig. 3).

### Histology confirms lower cellular ER stress and greater tissue preservation with exenatide treatment

**Fig. 4:**
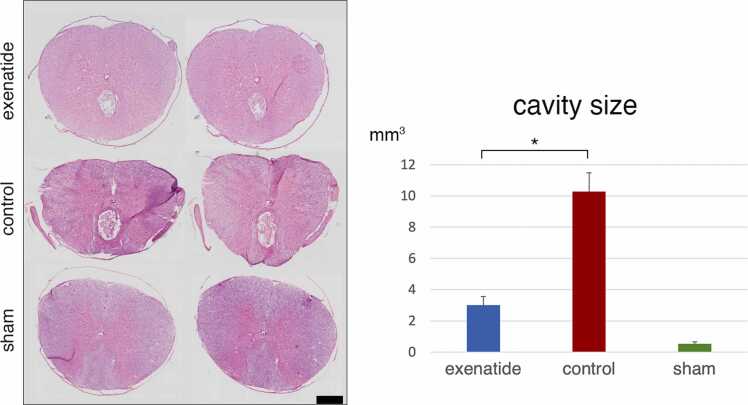
Hematoxylin-eosin staining reveals decreased cavity in the exenatide group. Sections taken 7 mm caudal to the lesion epicenter from spinal cords collected on day 14 after injury were stained with hematoxylin-eosin staining. Quantification of the cavitation area revealed significantly smaller lesions in the exenatide group (p < 0.05, n = 5 per group).

**Fig. 5:**
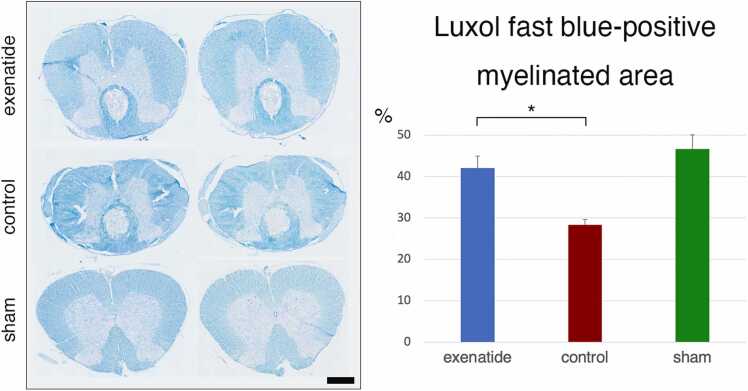
Luxol fast blue staining reveals higher residual myelination in the exenatide group. Sections taken 7 mm caudal to the lesion epicenter from spinal cords collected on day 14 after injury were stained with Luxol Fast Blue (LFB) and quantified. Compared with the control group, the exenatide group had significantly higher LFB-stained area, suggesting that exenatide administration mitigated the secondary injury process and led to decreased demyelination compared to the control group (p < 0.05, n = 5 per group).

The lesion cavity volume was evaluated with HE-stained sections from day 14 animals, revealing a significantly larger cavity in the control group compared to the exenatide group (Fig. 4). Myelination visualized with LFB staining was quantified, revealing significantly higher LFB-positive ratio in the exenatide group compared to the control group, suggesting that exenatide administration mitigated the secondary injury process and led to decreased demyelination compared to the control group (Fig. 5).

**Fig. 6:**
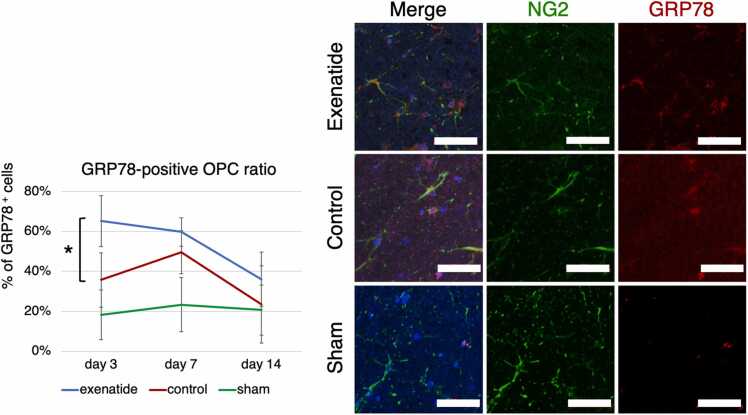
Exenatide increases GRP78 expression in oligodendrocyte precursor cells (OPCs). Quantification of NG2-positive OPCs in the dorsal column 7 mm caudal to the lesion epicenter showed that OPCs expressing GRP78 were significantly higher in the exenatide group on day 3 (p < 0.01, n = 5 per group).

**Fig. 7:**
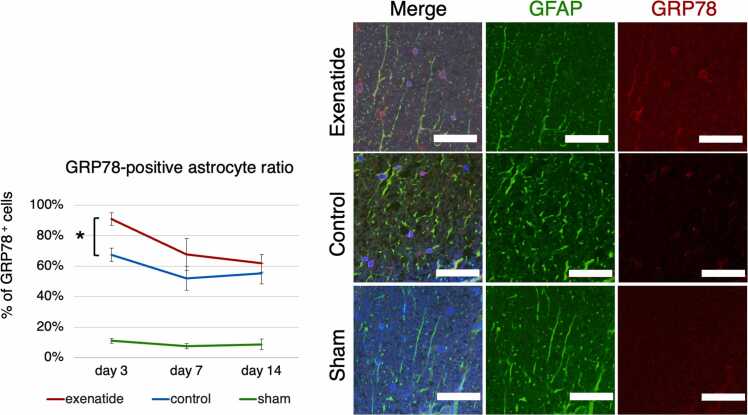
Exenatide increases GRP78 expression in astrocytes. Quantification of GFAP-positive astrocytes in the dorsal column 7 mm caudal to the lesion epicenter showed that astrocytes expressing GRP78 were significantly higher in the exenatide group on day 3 (p < 0.01, n = 5 per group).

**Fig. 8:**
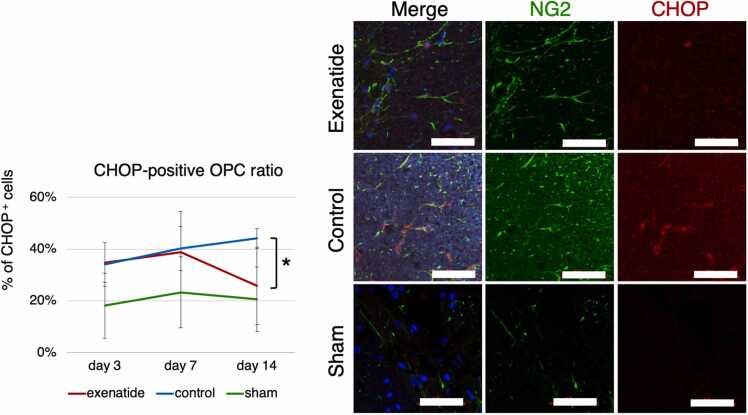
Exenatide decreases CHOP expression in oligodendrocyte precursor cells (OPCs). Quantification of NG2-positive OPCs in the dorsal column 7 mm caudal to the lesion epicenter showed that OPCs expressing CHOP were significantly lower in the exenatide group on day 3 (p < 0.01, n = 5 per group).

**Fig. 9:**
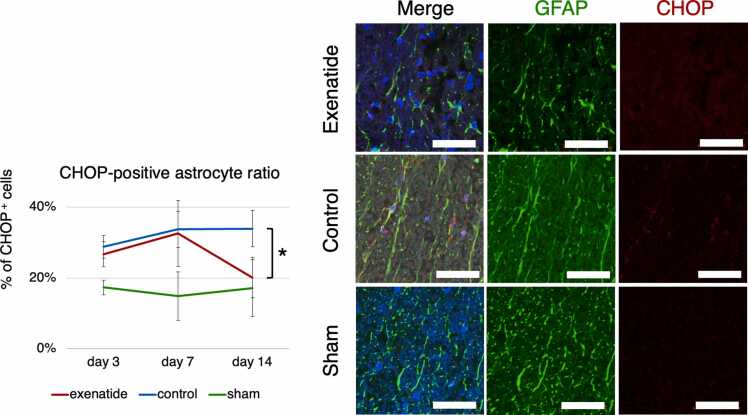
Exenatide decreases CHOP expression in astrocytes. Quantification of GFAP-positive astrocytes in the dorsal column 7 mm caudal to the lesion epicenter showed that astrocytes expressing CHOP were significantly lower in the exenatide group on day 3 (p < 0.01, n = 5 per group).

**Fig. 10:**
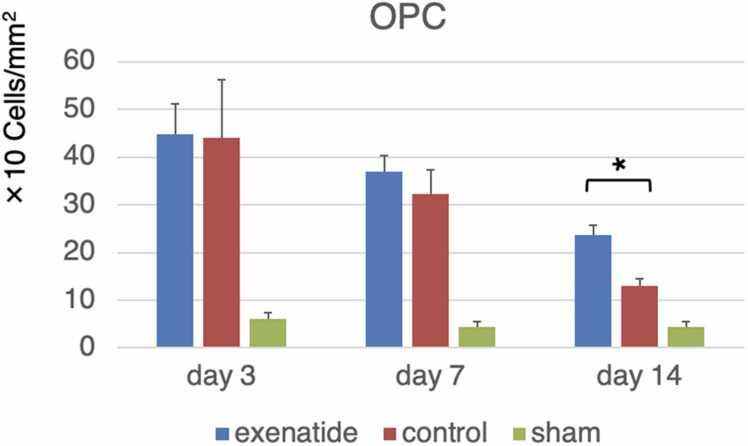
Quantification of oligodendrocyte precursor cells (OPCs) in the injured spinal cord reveals higher numbers in the exenatide group. The number of NG2-positive OPCs in the dorsal column 7 mm caudal to the lesion epicenter of five consecutive sections was manually quantified, revealing significantly higher number of OPCs in the dorsal column of the exenatide group (p < 0.05, n = 5 per group).

Cell-specific expression of the ER-stress indices was examined by immunohistochemistry. NG2-positive oligodendrocyte precursor cells and GFAP-positive astrocytes in the dorsal column that were also positive for GRP78 or CHOP were quantified. GRP78 immunoreactivity throughout the entire dorsal column tissue tended to be higher in the exenatide group at all time points, with a statistically higher ratio of positive cells in both OPCs and astrocytes of the exenatide group compared to the control group at day 3 (p < 0.01) (Fig. 6, Fig. 7). The expression of CHOP increased similarly in both groups from day 3 to day 7, but CHOP expression of the exenatide group was significantly lower compared to the control group in both OPCs and astrocytes at day 14 (p < 0.05) (Fig. 8, Fig. 9). The percentage of NG2-positive OPCs in all cells in the dorsal column was quantified, showing that OPCs increased after SCI in both the exenatide and control groups compared to the sham group. Although the percentage of OPCs decreased thereafter in both groups, it was higher in the exenatide group at all time points and was significantly higher in the exenatide group compared to the control group on day 14 (Fig. 10), suggesting that the administration of exenatide decreased the apoptosis of OPCs after SCI.

### Exenatide group demonstrated improved hindlimb motor function

**Fig. 11:**
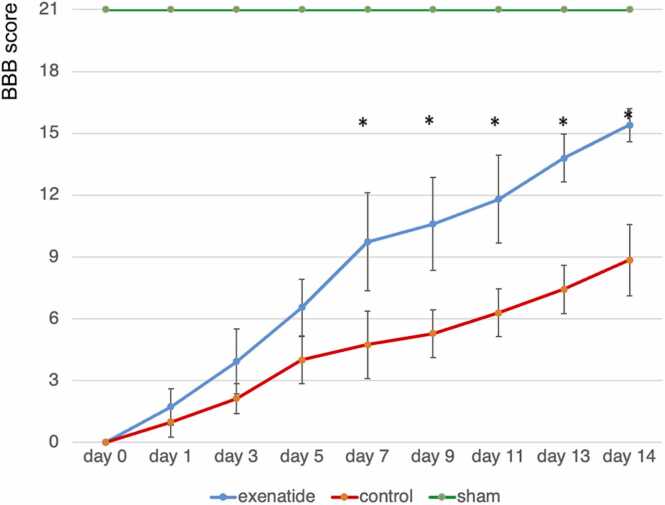
Hindlimb motor function reveals functional improvement in the exenatide group. BBB scores revealed significantly higher hindlimb motor scores in the exenatide group compared to the control group from day 7 and later (p < 0.01, n = 10 per group).

The BBB scale was 0 on the day after injury in both groups and subsequently improved over time. The exenatide treatment group showed a significant improvement in BBB score from day 7 onwards compared to the control group (p < 0.05), with the mean BBB scale at final observation on day 14 after injury being 15.4 in the exenatide treatment group while it was 8.9 in the control group (Fig. 11).

## Discussion

GLP-1 is a 30-amino acid peptide that is mainly secreted from intestinal endocrine L-cells to activate insulin production in pancreatic β-cells and lower blood glucose levels. GLP-1 functions by activating its specific receptor, the GLP-1 receptor (GLP-1R), and activation of the GLP-1R has been shown to have a wide range of effects, such as neuronal cell proliferation and differentiation, neurite outgrowth, and enhancement of synaptic plasticity (Erbil et al., 2019, Hakon et al., 2015, Hölscher, 2012, Li et al., 2012). GLP-1R activation in the brain exerts neurotrophic and neuroprotective effects, demonstrating protective effects against various neurodegenerative conditions such as ischemia, stroke, Alzheimer's disease, Parkinson's disease, and brain trauma (Fang et al., 2020) (Bader et al., 2019) (Rachmany et al., 2013). The results of this study confirm that exenatide, a GLP-1 receptor agonist, subcutaneously administered immediately after SCI and again 7 days later improves motor function recovery and suggests that part of this improvement is brought about by a reduction in ER stress.

One point of concern when using a drug to treat diabetes after SCI was the possibility of hypoglycemia. GLP-1 receptor agonists have been shown to secrete insulin mainly in hyperglycemic conditions and are generally considered unlikely to cause hypoglycemia. In this study, the exenatide group showed lower blood glucose levels compared to control animals, but the differences were not statistically significant. There may be several reasons that exenatide led only to a mild depression in blood glucose levels. First, hyperglycemia is often seen in SCI patients in clinical practice, even in patients without any history of diabetes. Considering that exenatide administration was immediately after injury, its effects may have been masked by this transient hyperglycemia. With the exacerbation of secondary damage and deterioration of motor function being reported in SCI of hyperglycemic model rats, it is anticipated that the antihyperglycemic effect of GLP-1 receptor agonists may also be beneficial to the recovery after SCI by reducing secondary damage. Furthermore, ER stress has been reported to be the driver of functional aggravation brought about by hyperglycemia after spinal cord injury, causing neuronal apoptosis and increasing blood-spinal cord barrier permeability, demyelination, and inflammation (Chen et al., 2019). Therefore, while the complete picture of the mechanisms through which exenatide improves motor function after SCI remains elusive, the reduction of ER stress and the prevention of hyperglycemia may be one benefit. Second, Khoo, et al. reported that exenatide administration to healthy volunteers revealed a mild decrease in fasting blood glucose but also brought about increased cortisol secretion that led to increased blood glucose levels during exercise (Kobayakawa et al., 2014). Since cortisol levels have been shown to increase immediately after SCI (Matsuyama et al., 2014), this may have countered the hypoglycemic effects of exenatide administration. Third may be related to exenatide dosage, which was derived from the clinical dosage for weekly exenatide adjusted for body weight. With many studies showing that the high metabolism of rodents requires a substantially higher dosage of drug to incite a similar action, the dosage of exenatide may have been too low to bring about hypoglycemia.

There have been a number of reports on the neuroprotective effects of GLP-1 receptor agonists, and the results suggest that various molecular mechanisms are involved. Exenatide has been shown to activate Schwann cells and promote nerve regeneration after peripheral nerve injury (Yamamoto et al., 2013). Although reports of exenatide in SCI are limited, there are several reports of the neuroprotective effects of exenatide or other GLP-1 agonists in murine SCI models. A report has shown that intraperitoneal administration of exenatide after SCI prevented mitochondrial cell death, improved hind limb function, and increased expression of myelin oligodendrocyte glycoprotein (MOG) (Durham-Lee et al., 2011). There has also been a report that intraperitoneal injection of exenatide after SCI in rats promoted autophagy, which decreased apoptosis in neurons and led to improved hindlimb motor function (Li et al., 2016). In that report a single intraperitoneal injection of exenatide led to significant motor improvement compared to control animals from as early as 7 days after administration. Our results confirmed improvement of motor function in a rat SCI model with subcutaneous exenatide administration that were comparable with intraperitoneal administration, demonstrating significant motor improvement from day 7 after administration. In this study, we decided to inject exenatide subcutaneously because that is the clinical route of administration. Regarding changes in administration methods, Parkes et al. reported on the pharmacokinetics of exenatide administered through multiple routes in rats and demonstrated that exenatide plasma levels after intraperitoneal and subcutaneous injections were elevated longer than that of intravenous doses, with subcutaneous injections showing higher plasma exenatide concentrations compared to intraperitoneal injections (Parkes et al., 2001).

Most likely in concert with the aforementioned mechanisms proposed for exenatide, our results suggest that a reduction of ER stress is a mechanism through which exenatide improves functional improvement after SCI. As outlined in the introduction, we have been researching the effects of ER stress in SCI and have previously shown how a reduction in ER stress using amiloride could rescue oligodendrocyte precursor cells (OPCs) from apoptosis, leading to an increased number of oligodendrocytes that can remyelinate neurons and improving hindlimb motor function. Contusive SCI has been shown to be predominantly a white matter injury, and we have focused our efforts on the oligodendrocyte lineage. This study is a preliminary exploratory project undertaken in order to probe whether or not exenatide affected ER stress levels within the injured spinal cord, and we posit that exenatide does in fact reduce ER stress. At the same time, it is important to acknowledge that SCI is a traumatic event that involves a wide variety of cellular stresses that activates the integrated stress response in which the ER stress response is a part of. We are currently performing follow-up studies looking into the molecular pathways involved in the ER stress response.

The involvement of ER stress in SCI as well as the changes seen in the three signal pathways Perk, ATF6, and IRE1a of the unfolded protein response has been well studied (Di Conza and Ho, 2020, Ghemrawi and Khair, 2020, Ohri et al., 2013, Ohri et al., 2012). In this preliminary study looking into changes in the post-SCI ER stress response after exenatide administration, we selected GRP78 as an index of general unfolded protein response to lower ER stress, and CHOP as a marker of severe ER stress that would initiate apoptotic signaling. In moderate thoracic contusive SCI, GRP78 and CHOP remained elevated for 1–12 h after SCI (Ohri et al., 2013), while their expression was higher and was maintained at high levels for the observed 72 h in severe SCI (Ohri et al., 2012). Studies observing these markers for a longer period have shown that these marker remained elevated for up to 14 days for both GRP78 and CHOP (Matsuyama et al., 2014). Considering that both short term and long term observational studies all report that the peak expression of both markers are in the first few days after SCI, we expected changes brought about by exenatide to become apparent during that acute period. While the significant increase in GRP78 in the exenatide group compared to the control group on day 3 is understandable, we cannot explain why a significant decrease in CHOP expression was observed in the exenatide compared to the control group on day 14. However, the fact that the immunohistochemistry results confirmed the time course of GRP78 and CHOP expression revealed by western blots makes it more likely that some kind of delayed response was initiated by exenatide administration. We plan to look into the expression of other unfolded protein response markers to look into this issue.

There are several limitation to this study. The greatest limitation stemmed from the short study period. This study was conducted to examine the short-term effects of exenatide administration on the recovery from SCI and has demonstrated significant molecular, histological, and behavioral improvements in the exenatide group compared to the control group. One limitation is the lack of multiple behavioral assessment tests. While the difference observed in hindlimb motor function is significant, the improvement would be more convincing if motor or sensory improvements were confirmed with another functional test. Another major limitation of this study is in the follow-up period of 14 days. The BBB data over the 14 days was not of sufficient length to follow the hindlimb motor function improvement from SCI until the animals of the two injured groups reached a plateau. Therefore, we cannot confirm if improvements seen in the exenatide group were maintained in the chronic phase after SCI. The study period was also not of sufficient duration to study the effects of exenatide on the oligodendrocyte lineage or its involvement in remyelination. This was an exploratory study to verify that exenatide administration after spinal cord injury improves motor function after SCI without severe side effects, and to research the possible involvement of ER stress in this process. We believe that the study period is sufficient to demonstrate that exenatide modulates ER stress, but its specific effects on the multiple pathways involved in the regulation of the UPR as well as the effects of this decreased ER stress will require longer study periods. Another limitation concerns our interpretation of the effect of exenatide on OPCs. We interpreted the increase of OPCs in the exenatide group as evidence of increased survival of the proliferating OPCs, but in fact we cannot exclude the fact that exenatide administration could increase the proliferation of OPCs or affect differentiation from local neural stem cells to favor the oligodendrocyte lineage. With the results of this study demonstrating significant improvement, our next step will be to study the long-term effects of exenatide as well as the dosage and administration schedule of exenatide.

## Conclusions

Combining the results of this study with previous reports on ER stress, it is thought that exenatide reduces secondary damage in the injured spinal cord by decreasing ER stress, thereby suppressing apoptosis, protecting myelin sheathes, and improving hind limb function after SCI. Past reports of exenatide administration after SCI have demonstrated that exenatide has multiple beneficial effects. The collective data indicate that exenatide enhances the mechanisms that regulate cell homeostasis, such as ER stress, mitochondrial stress, and autophagy, resulting in a net reduction in the secondary injury process. Further studies are needed to examine the full effects of exenatide on the ER stress response in the injured spinal cord as well as its effects on different cell types, including macrophages, mature oligodendrocytes, and neurons.

## Institutional review board statement

The Institutional Review Board of the Tokai University.

## Funding

This work was supported by 10.13039/501100001691JSPS KAKENHI Grant-in-Aid for Scientific Research (C) number 18K090984 and 20K09417.

## CRediT authorship contribution statement

**Satoshi Nomura:** Conceptualization, Methodology, Validation, Formal analysis, Resources, Data curation, Writing – original draft, Writing – review & editing, Visualization,. **Hiroyuki Katoh:** Conceptualization, Methodology, Writing – review & editing. **Sho Yanagisawa:** Methodology. **Toshihiro Noguchi:** Validation, Visualization. **Keiko Okada:** Validation, Visualization. **Masahiko Watanabe:** Project administration.

## Declaration of Competing Interest

The authors have not competing interests to declare.
